# The interplay between environmental exposures and COVID-19 risks in the health of children

**DOI:** 10.1186/s12940-021-00716-z

**Published:** 2021-03-26

**Authors:** Peter D. Sly, Brittany A. Trottier, Catherine M. Bulka, Stephania A. Cormier, Julius Fobil, Rebecca C. Fry, Kyoung-Woong Kim, Steven Kleeberger, Pushpam Kumar, Philip J. Landrigan, Karin C. Lodrop Carlsen, Antonio Pascale, Fernando Polack, Mathuros Ruchirawat, Heather J. Zar, William A. Suk

**Affiliations:** 1 Children’s Health and Environment Program, The University of Queensland, Brisbane, Australia; 2 Superfund Research Program, National Institute of Environmental Health Sciences, 530 Davis Drive, Durham, NC 27709 USA; 3 Department of Environmental Sciences and Engineering, University of North Carolina, Chapel Hill, USA; 4 LSU Superfund Research Program, Louisiana State University, Baton Rouge, USA; 5 Department of Biological, Environmental and Occupational Health Science, University of Ghana, Accra, Ghana; 6 School of Earth Sciences and Environmental Engineering, Gwangju Institute of Science and Technology, Gwangju, South Korea; 7 Immunity, Inflammation, and Disease Laboratory, National Institute of Environmental Health Sciences, Durham, USA; 8 United Nations Environment Program, Nairobi, Kenya; 9 Schiller Institute for Integrated Science and Society, Boston College, Chestnut Hill, USA; 10 Division of Paediatric and Adolescent Medicine, University of Oslo & Oslo University Hospital, Oslo, Norway; 11 Department of Toxicology, Faculty of Medicine, University of the Republic, Montevideo, Uruguay; 12 Fundación INFANT, Buenos Aires, Argentina; 13 Chulabhorn Research Institute, Bangkok, Thailand; 14 Dept of Paediatrics & Child Health and SA-MRC Unit on Child & Adolescent Health, University of Cape Town, Cape Town, South Africa

**Keywords:** Children’s environmental health, COVID-19, Combined exposures, SARS-Cov-2

## Abstract

**Background:**

An unusual feature of SARS-Cov-2 infection and the COVID-19 pandemic is that children are less severely affected than adults. This is especially paradoxical given the epidemiological links between poor air quality and increased COVID-19 severity in adults and that children are generally more vulnerable than adults to the adverse consequences of air pollution.

**Objectives:**

To identify gaps in knowledge about the factors that protect children from severe SARS-Cov-2 infection even in the face of air pollution, and to develop a transdisciplinary research strategy to address these gaps.

**Methods:**

An international group of researchers interested in children’s environmental health was invited to identify knowledge gaps and to develop research questions to close these gaps.

**Discussion:**

Key research questions identified include: what are the effects of SAR-Cov-2 infection during pregnancy on the developing fetus and child; what is the impact of age at infection and genetic susceptibility on disease severity; why do some children with COVID-19 infection develop toxic shock and Kawasaki-like symptoms; what are the impacts of toxic environmental exposures including poor air quality, chemical and metal exposures on innate immunity, especially in the respiratory epithelium; what is the possible role of a “dirty” environment in conveying protection – an example of the “hygiene hypothesis”; and what are the long term health effects of SARS-Cov-2 infection in early life.

**Conclusion:**

A concerted research effort by a multidisciplinary team of scientists is needed to understand the links between environmental exposures, especially air pollution and COVID-19. We call for specific research funding to encourage basic and clinical research to understand if/why exposure to environmental factors is associated with more severe disease, why children appear to be protected, and how innate immune responses may be involved. Lessons learned about SARS-Cov-2 infection in our children will help us to understand and reduce disease severity in adults, the opposite of the usual scenario.

## Background

The virus SARS-CoV-2 emerged in Wuhan, China and was reported to the World Health Organization (WHO) on December 21, 2019 [1]. WHO subsequently declared a Public Health Emergency of International concern on January 30, 2020 [1] and a pandemic on March 11, 2020 [2]. To date, the number of confirmed cases exceeds 110 million, with hundreds of thousands of deaths worldwide.

SARS-CoV-2 infects by binding to the angiotensin converting enzyme 2 (ACE2) receptor, which is expressed in several organs including lung, heart, kidney, and intestine [3]. Common symptoms of the disease, called COVID-19, appear 2–14 days after viral exposure, and may include: cough, dyspnoea, fever, chills, muscle pain, headache, sore throat, diarrhea, and loss of taste or smell [4].

### COVID-19 and health related societal benefits

Human health in general and children’s health in particular is a key element of the Inclusive Wealth of a nation; which is the sustainability of economy and wellbeing of their people and is measured by assessing natural capital, human capital, and produced capital [5]. Health is a critical piece of the human capital component of countries and is fundamental to countries’ well- being. Improvements in human health can increase the human capital component of Inclusive Wealth and benefit society in three main ways: by directly enhancing well-being, raising productivity and extending life years. Increased longevity is widely used as a proxy for increases in health-related human capital, because there are only limited options for accurately quantifying increased well-being (happiness) and productivity [6].

Capturing accurate information on children’s health, including environmental exposures that may be detrimental to their health, enables countries to track their national well-being. Health metrics, especially measures of children’s health, provide an inclusive measure of national progress and wellbeing that extends beyond GDP. There has been increasing demand for such information in the policy and research arenas in recent years [7–9]. Estimating the nexus between environmental exposures and COVID-19 and computing their cumulative impact on children’s health would provide critically important information on the impacts of the COVID-19 pandemic on progress toward the Sustainable Development Goals (SDGs), especially SDGs 1**,** 2 and 3, and on the societal benefits of healthy children.

### Covid-19 and children

Among children < 18 years with Covid-19 [10], the median age was 11 years (range 0–17 years). Nearly one third of reported pediatric cases (813; 32%) occurred in children aged 15–17 years, followed by those in children aged 10–14 years (682, 27%). Among younger children, 398 (15%) occurred in children aged < 1 year, 291 (11%) in children aged 1–4 years, and 388 (15%) in children aged 5–9 years.

Based on available evidence, children do not appear to be at high risk for severe COVID-19, with most infected children having asymptomatic or mild illness [11]. Emerging evidence shows, however, that a subset of children develop toxic shock and Kawasaki-like symptoms due to a systemic vasculitis [12]. How this syndrome may relate to the severe inflammatory condition with cytokine storm seen in some adults with COVID-19 is unclear. These observations raise three important questions: Why are children less seriously affected by SARS-CoV-2? Why do some children develop very severe COVID-19? What are the routes of children’s exposure to SARS-CoV-2 that may differ from adult exposures and alter their susceptibility?

Another important question is the role children play in community transmission of SARS-CoV-2 [13]. One intriguing, but small study, suggests that children may have higher viral loads despite having fewer symptoms that adults with severe disease [14]. If this observation is substantiated in larger studies, the reasons why need thorough investigation.

A recent analysis of the WHO Global Research Roadmap on COVID-19 [15], while comprehensive overall, did not address children, or seek to understand why children are apparently less severely affected by COVID-19. To address this oversight, we have chosen to specifically focus on children and to identify knowledge gaps and research opportunities in this area.

### Air pollution and COVID-19

Epidemiological evidence suggests environmental exposures influence the occurrence and severity of COVID-19. Other comorbid factors may contribute additionally to inter-individual variation in response to respiratory infections such as SARS-CoV-2, including gender, age, socioeconomic status, nutrition, pre-existing conditions or diseases, and genetic predisposition. A genetic contribution to human disease susceptibility has been demonstrated for many viruses, and thus likely will be important in responses to SARS-CoV-2 infection [16, 17]. There are clear gaps in our knowledge related to why children have less severe COVID-19 disease. These gaps provide opportunities for specific research.

Air pollution is an example of an environmental exposure influencing the occurrence and severity of COVID-19; areas in the USA with higher levels of air pollution have higher incidence and mortality rates from COVID-19; with every 1 μg/m^3^ increase of PM_2.5_ associated with an 8% increase in risk of death [18]. While more research is needed, there are a number of plausible explanations for the air pollution phenomenon worth exploring, including: pollution induced oxidative stress impairing respiratory epithelial barrier function [19], reducing innate immune anti-viral responses [20] or impairing macrophage function [21, 22]; inducing an immune suppression microenvironment in the lungs [23–26]; pollution-induced inflammation resulting in more severe COVID disease [27]; and the intriguing possibility that PM_2.5_ may carry SARS-CoV-2 deeper into the lungs [28]. On theoretical grounds oxidants in air pollution could induce DNA damage in the virus, altering its genome and increasing infectivity or enhancing pathogenesis. Exposure to other environmental toxicants could also impair host responses, with a growing list of toxicants associated with impaired vaccine responses in children [29]. Air pollution is known to modify innate immune responses initiated via toll-like receptors [30]. However, the association with pollution and severe Covid-19 is not seen to the same extent in impoverished environments and, as will be discussed later, raises important research questions.

Children are more susceptible than adults to the adverse health effects of pollution [31, 32], yet are less susceptible to severe COVID-19. Does this mean that the increasing evidence linking air pollution to severe COVID-19 is specific for adults or is something protecting children? It would seem there may be different mechanisms underlying the interaction between COVID-19 infection and air pollution in children and in adults. It may be hypothesized that developmental mechanisms, such as higher levels of ACE2 expression, especially in the lungs, prior non-SARS-CoV-2 corona virus infections, innate immune “training”, intrinsic elevation of lymphocytes, including T, B, and NK cells, [33], or a suppressive/Th2 skewed immune microenvironment (preventing a strong immune and inflammatory response in the lungs) may protect children from severe disease [34].

### Research needs

A recent workshop organised by the National Academies of Sciences (NAS) on the impact of environmental exposures on infectious diseases highlighted the need for integrated, trans-disciplinary research in this area. Speakers in the workshop discussed how global trends and interconnections demonstrate a need for environmental health and infectious disease communities to converge but also a need for diverse fields to be a part of the conversation as well [35]. Quoting from their report “Emerging findings suggest that exposure to environmental pollutants such as airborne particulate matter, industrial chemicals, and heavy metals may alter the immune system, increasing human susceptibility to infection” [35]. New research findings show that the microbiome of humans and ecosystems also play important roles in infection.

Despite this powerful call for trans-disciplinary research, the fields of environmental health and infectious disease are largely distinct from one another even though research on the interplay between these fields could inform new health practices, public health research, and public health policy [35]. In this commentary we take the NAS call for trans-disciplinary research one step further by highlighting the combined impacts of infections and environmental exposures impacts on children’s health. A new research agenda is required to address these questions. We have identified gaps in knowledge that need urgent research attention.

#### SARS-CoV-2 infection during fetal development

Pregnant women and the developing foetus are known to highly vulnerable to certain viral infections such as rubella and Zika virus, but is this the case for SARS-CoV-2? While limited data are available, information suggests evidence of vertical transmission among approximately 3% of mothers with COVID [36] and no adverse effects on the newborn [37]. Research is needed to understand why. Suggestions have been made that the normal Th-1 bias of the developing fetal immune system may be protective [38]. Alternatively, the potential for SARS-CoV-2 to induce an imbalanced T-reg/TH-17 response could pose a threat to the developing fetus [39]. Maternal exposure to air pollution containing strong oxidants, such as environmentally persistent free radicals (EPFRs) results in delayed maturation of Th-1 immune response postnatally [40]. A better understanding that incorporates an interdisciplinary approach utilizing both epidemiological and mechanistic research programs will be required to understand the true impact of SARS-CoV-2 on pregnant women and their developing fetuses.

#### Age and genetic susceptibility to severe Covid-19

Severe COVID-19 in adults is characterised by an acute cytokine storm associated with a severe acute respiratory distress syndrome (ARDS) leading to multiple organ dysfunction and death from respiratory failure [41]. However, under this large umbrella of COVID-19 there are many endotypes that suggest multiple disease mechanisms. There is little understanding of why some patients develop severe disease and die, some develop mild disease and others appear to remain as asymptomatic carriers. Recent studies postulate that telomere length in leukocytes might serve to identify patients more likely to die from SARS-Cov-2 infection, regardless of age. Telomere shortening in leukocytes has been associated with increased synthesis of pro-inflammatory cytokines [42, 43] that lead to severe disease. However, further investigation on the use of telomere length as a biomarker for cytokine response to SARS-Cov-2 infection is needed. Telomeres shorten with cell division but whether a systematic difference of telomere length, as described between children and adults [44] could contribute to the differences in inflammatory response and disease severity with COVID-19 is unknown.

Individual genetic susceptibility is likely to have a role in severity of disease after infection with SARS-CoV-2 [16, 17] and may also influence viral infectivity and vaccine efficacy, as has been demonstrated for other viral infections [45]. Why a severe cytokine storm does not appear frequently in children remains a mystery, especially when this can occur in other childhood conditions. The “macrophage activation syndrome” is a potentially life-threatening condition seen in some children with systemic juvenile idiopathic arthritis [46]. Why does this not occur with SARS-CoV-2?

#### Previous infection with corona viruses

Children are frequently infected with endemic seasonal coronaviruses. In a community-based observational study in which parents took nasal swabs from healthy infants weekly for the first 2 years of life [47], swabs were analysed for multiple viruses and bacteria, including four endemic corona viruses. Infection with corona virus was common, with 11.5% of children experiencing their first infection by 6 months of age, 33.4% by 12 months, 52.9% by 18 months and 72.2% by 2 years of age [48]. Most first infections were associated with respiratory symptoms, but 20% were asymptomatic [48]. Does the common occurrence of mild or asymptomatic infections with relatively innocuous corona viruses protect children from infection or from severe disease with SARS-CoV-2?

#### Why does air pollution increase disease severity and mortality in adults but not in children?

As outlined above, there is an epidemiological association between increased levels of air pollution, especially levels of PM_2.5_, and severe COVID-19 with increased mortality. While various theories abound, PM is known to act as a carrier for viruses and bacteria [28]. This effect has been seen in adults but not in children. There is a clear gap in knowledge and research priority in this area to explain this observation as children are generally much more vulnerable to the toxic effects of poor air quality [31, 32]. Why this is not the case with SARS-Cov-2 infection is a mystery that needs to be solved.

#### Impact of environmental chemical exposures and Covid-19 on the respiratory system

Children are more vulnerable than adults to the adverse effects of pesticides and many other toxic chemicals via multiple mechanisms including: route of exposure; physiological differences that make children more susceptible; and the immaturity of organ systems during fetal development and childhood [49]. Children are at increased risk of acute poisonings, chronic toxicity, and long-term effects [49]. The respiratory system, as a target of SARS-Cov-2, can be affected by environmental chemicals, either through inhalation exposure or via systemic toxicity. Environmental exposure to several toxicants has been associated with a higher risk of respiratory disease, as well as with alterations of the immune system that could predispose to a greater risk of acquiring the SARS-Cov-2 infection as well as a worse prognosis of other infectious diseases. A large number of chemical disinfectants, including alcohols, chlorine compounds, formaldehyde, and quaternary ammonium compounds, are commonly used in household settings. The inappropriate and / or indiscriminate use of disinfectants in domestic settings may also increase the risk of respiratory symptoms and clinical manifestations secondary to their irritant effects [50].

Acute or chronic low-level inhalation of ***pesticides*** may exacerbate asthma or increase the risk of developing asthma, through the interaction with functional irritant receptors in the airway and promoting neurogenic inflammation. Organophosphate insecticides cause airway hyper-reactivity via a common mechanism of disrupting negative feedback control of cholinergic regulation in the lungs. Synergism with allergen sensitization has also been reported, particularly with fungicides exposure [51].

Exposures to ***organic chemicals*** could also indirectly influence the severity of viral infections by increasing the risk for chronic conditions that worsen COVID-19 prognoses [52]. Among the most common comorbidities are obesity, hypertension, and diabetes, conditions that have each been attributed to organic chemical exposures [53]. Several industrial chemicals recognized for their endocrine disrupting potential have been labeled as “obesogens” for their impacts on lipid metabolism and adipogenesis, and in turn, weight gain [54, 55]. While obesity may not be solely explained by organic chemical exposures, a deeper understanding of their indirect role in the COVID-19 pandemic is needed.

#### Do environmental chemical exposures alter innate immune anti-viral responses to contribute to move severe disease?

Exposure to environmental toxicants (e.g., *pesticides, heavy metals, endocrine-disrupting chemicals, among others*) have been linked with alterations in the immune system [56, 57]. Possible consequences of immunotoxicants related to infectious disease may include increased susceptibility to infection, prolonged recovery periods, and reduced responses to vaccinations [58]. Prenatal exposure to dioxin like compounds may cause postnatal immune dysfunction and increased susceptibility to infectious and allergic diseases in childhood [59].

Exposure to organochlorines compounds, organophosphates, carbamates and several herbicides have been associated with immunotoxicity. Such pesticides can induce release of pro-inflammatory mediators (TNF-α, IL-1β, IL-6) from macrophages, increase aromatase expression, induce oxidative stress, have estrogenic activity resulting in endocrine disruption, and may induce DNA damage acting as direct carcinogens [60]. Heavy metals exposure (particularly lead) has been associated with toxic effects on the immune system and a higher risk of inflammatory disorders; increasing the incidence of infectious disease, autoimmunity and cancer [56]. The immunotoxic effects of lead are complex, with some data suggesting preferential promotion of Th-2 development by suppressing Th-1 cell proliferation [61]. What effect this has on immune development in early life is unknown. A wide variety of environmental toxicants can impair a protective immune response, especially in organs expressing high ACE2 expressions, such as intestine and kidney, via interactions with the aryl hydrocarbon receptor (AhR) [60]. Whether children are more or less vulnerable to environmental activation of the AhR is not known.

***Toxic metals*** are ubiquitous in the environment as a result of both geogenic and anthropogenic sources. Epidemiologic studies of arsenic exposure in relation to established viral infections highlight its potential to increase infection susceptibility. Cross-sectional analyses of both children and adults enrolled in the National Health and Nutrition Examination Survey (NHANES) have observed that higher urinary total arsenic measurements (i.e., the sum of organic and inorganic forms) are associated with a higher prevalence of hepatitis viruses A and B and higher rates of varicella zoster virus reactivation [29, 62, 63]. Other research suggests pregnancy may be a particularly vulnerable window for the immunotoxicity of inorganic arsenic (iAs). For example, in a prospective study, women with higher urinary iAs concentrations throughout pregnancy were more likely to seroconvert to hepatitis E virus [64]. The risk of iAs-induced immunotoxicity during pregnancy appears to affect both the mother and the offspring. Data collected by the New Hampshire Birth Cohort Study show in utero exposures to iAs and its metabolites are associated with a higher risk of various infections throughout infancy [65–67]. The precise biological mechanisms by which iAs increases the risk for infectious disease are incompletely understood but may be related to impaired function of macrophages, the leukocyte responsible for patrolling for and destroying pathogens [68]. In a case-control study of a population with arsenical skin lesions, investigators found the macrophages of iAs-exposed individuals had reduced phagocytic capacity and reduced bacterial killing [69]. Taken together, these data suggest that iAs has immunosuppressive activity with the potential for chronic exposures to increase susceptibility to viral infections. The impact of As exposure on prevalence and severity of COVID-19, especially on children, is unknown. However, given the widespread exposure to arsenic globally and the impact on other viral infections, this certainly warrants investigation.

Environmental exposure to other toxic metals including cadmium (Cd), lead (Pb), and mercury (Hg) decrease immune responses and increased the risk of infectious diseases [70–72]. For example, prenatal exposure to Hg (28–30 weeks gestation) was associated with increased risk of lower respiratory tract infections when the infants were between 9 to 12 months of age [71]. In a cross-sectional study of non-smokers, who tend to have relatively low toxic metal exposures, blood Cd and Pb levels were associated with prevalent hepatitis B infections [72]. A currently understudied area in relation to COVID-19 risk is the impact of metals mixtures. There is a strong possibility that metals interact with one another to a have a synergistic or antagonistic effect on the immune system, including susceptibility to infections. Given the ubiquitous exposure to toxic metals in the environment, research into how mixture-based exposures could increase susceptibility to COVID-19, especially in children, is warranted.

***Organic compounds*** including; polychlorinated biphenyls (PCBs), dioxins, pesticides, and per and polyfluoroalkyl substances (PFAS), are known to impair immune responses [73–77]. Epidemiologic research has linked prenatal PFAS exposures to an increased risk of lower respiratory tract infections, common cold episodes, and other infections later in childhood [78–80]. Likewise, prenatal exposures to PCBs and dioxins have been implicated with upper respiratory tract infections during infancy [81]. The immunotoxic mechanisms through which organic chemicals increase susceptibility to infections are unknown but may be due to reducing immune responses to infectious agents. Exposures that reduce anti-viral innate immune responses could, in theory, increase the risk of COVID-19, especially in children where anti-viral responses are already low.

#### Will exposure to environmental toxicants reduce the effectiveness of vaccines against SARS-Cov-2?

Exposures to organic chemicals including dioxins, PCBs, and PFAS may also diminish vaccine effectiveness. In a study of mother-child dyads enrolled in the Norwegian Mother and Child Cohort (MoBa), maternal exposure to PCBs and dioxins during pregnancy was associated with reduced antibody responses to the measles vaccine in offspring at age 3 [81]. Other studies from the Faroe Islands suggest that associations of PCB exposures with vaccine responses depend on the timing of exposures [82, 83]. For instance, when researchers compared the effects of prenatal PCBs to levels at the age of 18 months, they found that the latter were more strongly associated with reductions in diphtheria toxoid antibody concentrations [83]. The researchers posited that the combination of intrauterine and breast milk exposures increased PCB body burdens at a time when the immune system is vulnerable [83]. Exposure timing also appears to be important for vaccine responses among individuals exposed to PFAS. In a study of healthy adults from New York immunized with FluMist, a live attenuated vaccine administered by intranasal spray for the prevention of influenza virus, no differences were observed in antibody responses by serum PFOA concentrations [84]. However, PFOA-exposed infants have been found to have reduced antibodies to tetanus and diphtheria at the age of 5 [85]. Overall, these findings show the potential for organic chemicals to suppress antibody production in response to vaccinations. Scientists should be aware that an eventual SARS-Cov-2 vaccine may not be as effective as intended in individuals exposed to organic chemicals.

#### What are the long-term consequences of COVID-19?

There are increasing reports of persistent symptoms after recovery from COVID-19 including fatigue, dyspnea, “brain fog”, joint pain, and chest pain [86] which raises the questions, what are the long-term health consequences of surviving Covid-19? Will long term health consequences only be seen in those with the most severe initial disease? Doctors are reporting a growing number of COVID-19 patients exhibiting signs of heart and neurological damage and it is unknown as to whether the damage will persist throughout life [87]. Research in this area is of critical importance for prevention and intervention activities as lasting health effects of children surviving COVID-19 could lead to significant health and economic costs throughout their lifespan, similar to the costs of managing chronic diseases. It is estimated that heart disease and stroke cost US healthcare systems $199 billion per year and causes $131 billion in lost productivity [88], a number that is sure to increase should COVID-19 induce chronic health effects in both children and adults. It is imperative that researchers consider environmental exposures in these studies as exposure to environmental chemicals has been linked to the development or exacerbation of chronic diseases and could increase the risk of developing long-term adverse health effects after SARS-Cov-2 infection and recovery. For example, research has shown that arsenic exposure induces epigenetic changes that can disrupt the endocrine system [89] as well as other research pointing to an association between arsenic exposure and lung disease [90] and hypertension [91]. Caballero et al. [92] also found that disease severity in infants after respiratory syncytial virus (RSV) infection was dependent on the interaction between exposure to environmental endotoxin and genetic background (i.e. gene x environment interaction). The interplay between the environment and the SARS-Cov-2 virus will be important to disentangle to develop the proper public health intervention and prevention activities.

#### Covid-19 in resource-limited settings: does a “dirty” environment protect children against COVID-19 – the hygiene hypothesis in action?

Similar to the pattern seen in high income countries, children develop predominantly mild or asymptomatic SARS-Cov2 infection in low and middle income countries (LMIC) settings [93]. Whereas lower respiratory tract infection (including from RSV, influenza or other viruses) remains a major killer in children under 5 years in LMICs with malnourished or immunosuppressed children particularly at risk, this pattern has not occurred with SARS-CoV-2 infection. Reasons for this are poorly understood, but a key hypothesis is that prior infection with other pathogens or universal BCG vaccination, may possibly protect children against severe disease through development of “trained” innate immunity [94]. Prior infection with seasonal coronaviruses which are endemic in LMICs and ubiquitous in children, may provide cross protection to SARS-CoV-2 from high levels of antibodies or from cellular immunity; pre-existing cross-reactive cellular immune responses to SARS-CoV-2, due to prior infection with seasonal coronavirus, have been shown that may protect against COVID-19 [95].

## Conclusion

A concerted research effort is needed to better understand the links between environmental exposures and COVID-19, especially air pollution and COVID-19. We call for specific research funding to encourage basic and clinical research to understand if/why exposure to environmental factors is associated with more severe disease, why children appear to be protected, and how innate immune responses may be involved. These research activities will be critical to develop prevention and intervention strategies for combined exposures as we are experiencing with COVID-19, to reduce the burden of disease. It will also provide much needed rationale for investment in public health and pollution management sectors. We need to learn the lessons offered by our children to understand how to reduce disease severity in adults, which is quite a change from usual scenarios.

## Data Availability

n/a

## Abbreviations

ACE2: Angiotensin converting enzyme 2
ARDS: Acute respiratory distress syndrome
Cd: Cadmium
EPFRs: Environmentally persistent free radicals
GDP: Gross domestic product
Hg: Mercury
iAs: Inorganic arsenic
LMIC: Low- and middle-income countries
MoBa: Norwegian Mother and Child Cohort
NAS: National Academies of Sciences
NHANES: National Health and Nutrition Examination Survey
NK cell: Natural killer cell
Pb: Lead
PCB: Polychlorinated biphenyl
PFAS: Per and polyfluoroalkyl substances
PM: Particulate matter
RSV: Respiratory syncytial virus
SDG: Sustainable Development Goal
WHO: World Health Organization
